# Antifungal and Surface Properties of Chitosan-Salts Modified PMMA Denture Base Material

**DOI:** 10.3390/molecules25245899

**Published:** 2020-12-13

**Authors:** Katarzyna Walczak, Georg Schierz, Sabine Basche, Carola Petto, Klaus Boening, Mieszko Wieckiewicz

**Affiliations:** 1 Department of Prosthetic Dentistry, Carl Gustav Carus Faculty of Medicine, Technische Universität Dresden, Fetscherstr. 74, 01307 Dresden, Germany; Georg.Schierz@mailbox.tu-dresden.de (G.S.); Klaus.Boening@uniklinikum-dresden.de (K.B.); 2 Clinic of Operative and Pediatric Dentistry, Carl Gustav Carus Faculty of Medicine, Technische Universität Dresden, Fetscherstr. 74, 01307 Dresden, Germany; Sabine.Basche@uniklinikum-dresden.de; 3 Department of Oral and Maxillofacial Surgery, Carl Gustav Carus Faculty of Medicine, Technische Universität Dresden, Fetscherstr. 74, 01307 Dresden, Germany; Carola.Petto@uniklinikum-dresden.de; 4 Department of Experimental Dentistry, Faculty of Dentistry, Wroclaw Medical University, 26 Krakowska st., 50-425 Wroclaw, Poland; m.wieckiewicz@onet.pl

**Keywords:** chitosan, chitosan glutamate, chitosan-HCl, antifungal, *C. albicans*, PMMA, denture base

## Abstract

Chitosan (CS) and its derivatives show antimicrobial properties. This is of interest in preventing and treating denture stomatitis, which can be caused by fungi. Therefore, the aim of this study was the development of a novel antifungal denture base material by modifying polymethyl methacrylate (PMMA) with CS-salt and characterizing its antifungal and surface properties in vitro. For this purpose, the antifungal effect of chitosan-hydrochloride (CS-HCl) or chitosan-glutamate (CS-G) as solutions in different concentrations was determined. To obtain modified PMMA resin specimens, the CS-salts were added to the PMMA before polymerization. The roughness of these specimens was measured by contact profilometry. For the evaluation of the antifungal properties of the CS-salt modified resins, a *C. albicans* biofilm assay on the specimens was performed. As solutions, both the CS-G and CS-HCl-salt had an antifungal effect and inhibited *C. albicans* growth in a dose-dependent manner. In contrast, CS-salt modified PMMA resins showed no significant reduced *C. albicans* biofilm formation. Furthermore, the addition of CS-salts to PMMA significantly increased the surface roughness of the specimens. This study shows that despite the antifungal effect of CS-salts in solution, a modification of PMMA resin with these CS-salts does not improve the antifungal properties of PMMA denture base material.

## 1. Introduction

Fungal infections show increased prevalence worldwide [1]. *Candida* spp. have several virulence factors promoting the colonization and opportunistic infection of host organisms by appropriate conditions [1]. *Candida* can adhere and grow on different surfaces, e.g., biomaterials as denture base materials. In general, adhesion of proteins and microorganisms, e.g., *Candida* to biomaterials depends on several factors such as surface charge, surface free energy, polarity, hydrophobicity, morphology, and roughness, as well as the composition of biomaterials. [1,2]. The acquired oral pellicle covers tissues and dental materials in the oral cavity [2]. It functions, e.g., as a protective layer and contains antibacterial components. On the other hand, it promotes the adherence of microorganisms and biofilm formation and changes the surface physiochemical properties [2].

Oral biofilm is related to caries, gingivitis, periodontitis, peri-implantitis, and denture stomatitis. The modulation of its formation in the oral cavity is challenging [2,3]. Thus, different anti-adherent, antimicrobial and antibiofilm strategies to improve oral biomaterials are studied [3,4].

Among the *Candida* spp. *C. albicans* is the predominant isolate from oral mucosal infections, e.g., denture stomatitis (DS) [1]. DS is a common mucosal disorder among denture wearers with a prevalence of up to 70% and is characterized by inflammation, erythema, and/or hyperplasia of the oral mucosa [5,6]. It is multifactorial and associated with denture biofilms, poor denture hygiene, poor denture quality, and nocturnal denture use [5,6]. As for other oral mucosal infections, *C. albicans* is the most prevalent and predominant *Candida* spp. associated with DS [6]. The leading etiological factor of DS is a pathogenic overgrowth of *Candida* on the oral mucosa and denture surface [6]. Thus, antifungal therapy is a key factor in the treatment of DS [6]. Nevertheless, the therapy lacks a gold standard and clinical strategies involve multiple approaches targeting biofilm formation and fungal infections of oral tissue [5]. One of the concepts is the topical application of oral antifungal substances such as miconazole, nystatin, or others [5], but therapeutic results depend highly on patient compliance [7]. Thus, the effectiveness of DS therapies is limited, and rapid recurrence of DS after topical antifungal therapy is common [6], mostly by yeast that survived and resists the treatment on the denture surface or in biofilm residues [6]. In particular, *Candida* biofilms show the ability to adhere on denture base material surfaces and form biofilms deeply embedded in cracks and imperfections that are hardly reachable by antifungals [6,8].

Polymers, especially polymethyl methacrylate (PMMA) are commonly used as denture base materials [9,10]. The advantages of PMMA are biocompatibility, low toxicity, and reliability [11]. The big disadvantage of PMMA is its susceptibility to biofilm formation [10]. Thus, concepts to enhance PMMA antimicrobial properties have been studied [10].

Because of the disadvantages described, research approaches to enhance the antifungal activity of dentures by mixing various antifungal substances to denture base materials are studied.

Organic antifungal agents (as nystatin, amphotericin, azole group derivatives), chlorhexidine digluconate, different inorganic particles (as silver nanoparticles, photo-catalysts, metallic oxides) or natural and herbal substances (thymoquinone, neem powder, tea tree oil, and others) are described [12,13,14,15,16,17]. The use of organic topical antifungals can result in the emergence of resistant strains, causing high costs and adverse effects [16,17]. Thus, natural products are seen as alternatives that are cheap, readily available, of local origin, and with minimal side effects [16]. Nevertheless, there is insufficient evidence for the use of natural products for the treatment of DS [17]. Thus, studies with novel, biocompatible, non-toxic, and effective natural products such as chitosan (CS) and its derivatives are needed.

Chitosan (CS) is a biopolymer [a (1→4) 2-amino-2-deoxy-β-D-glucan] experiencing increased attention in dental material science, especially due to its antimicrobial and hemostatic properties [18,19,20,21,22,23]. CS is mostly produced from chitin by N-deacetylation [21,24]. The biggest source of chitin is the shell waste of crustaceans from the fishing industry [21,24]. CS has reactive amino and hydroxyl groups and acts as a basic cationic polyelectrolyte after protonation of the amine groups [21,24]. CS is soluble in organic diluted acids [21,24,25]. To increase their water solubility, CS derivatives can be synthesized [21,22]. The products of the reaction of CS with acids are water-soluble CS-salts such as CS-hydrochloride, CS-acetate, CS-glutamate [23,25,26]. CS is non-toxic, biocompatible and biodegradable and can be applied as a biomaterial [18,21,22,24,27]. The biocompatibility of CS derivatives depends on their linked groups [21]. The antimicrobial activity of CS was evaluated in many studies with the general consensus being that it depends on many factors, e.g., microorganism type, chitosan source, chemical and physical formulation, environmental properties [18,22]. Furthermore, nonspecific antifungal (*Candida* spp.) effects of CS due to the inhibition of fungal cell adherence to host cells are described [28].

In vitro studies showed the antifungal (*Candida* spp.) activity of CS nanoparticles as a free solution [29], such as dispersed in tissue conditioner fluid [30], and incorporated into light-cured dental resin [31] as well as for CS incorporated into tissue conditioner [32]. Furthermore, the high molecular weight of CS decreases the hydrophobicity and adhesion of *C. albicans* to epithelial and human fibroblast cells [33] and the adhesion to dentures [34]. Furthermore, an in vivo study showed promising results for the use of low-molecular weight (LMW) CS solution for the treatment of DS [35]. Therapy with CS solution decreased the erythematous surface area, burning sensation, the time required for clinical improvement, and the number of blastospores and mycelia [35]. The results were comparable to nystatin treatment [35]. Moreover, Mustafa et al. showed the good antifungal efficacy of CS and CS–curcuminoid mouthwash in their randomized clinical trial [36].

In a previous pilot study (own unpublished data), the authors investigated the mechanical properties of PMMA modified with CS powder (Chitoscience 90/500). The mechanical properties of the modified material decreased, due to the large powder grain size of the chitosan used. Chitosan-hydrochloride (CS-HCl) and chitosan-glutamate (CS-G) can be easily purchased in smaller grain sizes. Both have antimicrobial activity: CS-HCl against *C. albicans* [37], and CS-G against Gram-positive and Gram-negative microorganisms [38]. Therefore, both types of CS-salts could be promising antimicrobial additives to PMMA.

The aim of this study was to develop a novel antifungal denture base material modified with CS-salts and to characterize its antifungal and surface properties in vitro. For this purpose, the antifungal effect of chitosan-hydrochloride (CS-HCl) or chitosan-glutamate (CS-G) as salt solutions in different concentrations was determined. For the evaluation of the antifungal properties of the CS-salt modified resins, a *C. albicans* biofilm assay on the specimens was performed. Furthermore, the roughness of these specimens was measured by contact profilometry.

The following null hypotheses were stated:

- CS-salts do not influence fungal cell growth,
- the fungal cell counts on CS-salt-modified denture base material do not differ from unmodified standard material (control),
- the roughness (Ra) of CS-salt-modified denture base material does not differ from unmodified standard material.

## 2. Results

### 2.1. Antifungal Test: Effect of Chitosan-Salt Solutions

**Figure 1:**
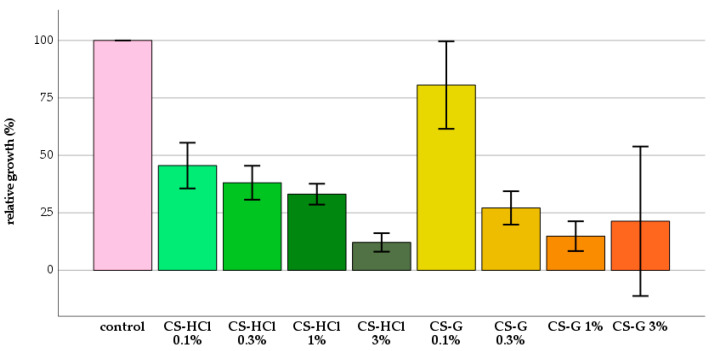
Mean relative growth (%) in each group, confidence interval (CI) 95%; chitosan glutamate (CS-G), chitosan-hydrochloride (CS-HCl).

Before the analysis of the antifungal properties of polymethyl methacrylate (PMMA) resins modified with chitosan (CS)-salts, the antifungal capacity of each CS-salt was determined. The analysis of relative growth showed that there was a good antifungal effect of both CS-salts. The relative fungal growth was decreased by CS-salts significantly in all groups compared to the control group (Figure 1).

### 2.2. Roughness 

**Figure 2:**
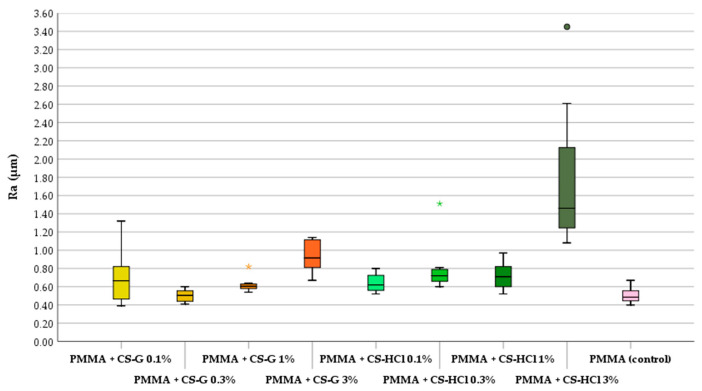
Roughness (Ra) in each group (box plot: median of mean roughness, 25% and 75% quartiles, * and ● indicate extremes); polymethylmethacrylate (PMMA), chitosan glutamate (CS-G), chitosan-hydrochloride (CS-HCl).

CS-salt powder particles can be identified on the specimen’s surface. This changes the aesthetic appearance of the material. The amount of CS-salt particles increases with a rising concentration of CS-salts added to the PMMA. The results of the contact profilometry showed that the surface roughness increased by adding CS-salts to the denture resin material (Figure 2). A significant increase in roughness (*p* ≤ 0.05) compared to the unmodified material was found when adding 1%, 3% CS-G and 0.1%, 0.3%, 1% and 3% CS-HCl to the PMMA material.

### 2.3. Antifungal Test: Effect of CS-Salt Modified PMMA 

The lowest *C. albicans* cell counts per CS-salt group were found on specimens modified with 1% of CS-HCl and with 1% of CS-G. The highest number of fungal cells per CS-salt was counted on specimens modified with 3% of CS-HCl and with 3% of CS-G (Table 1). 

**Figure 3:**
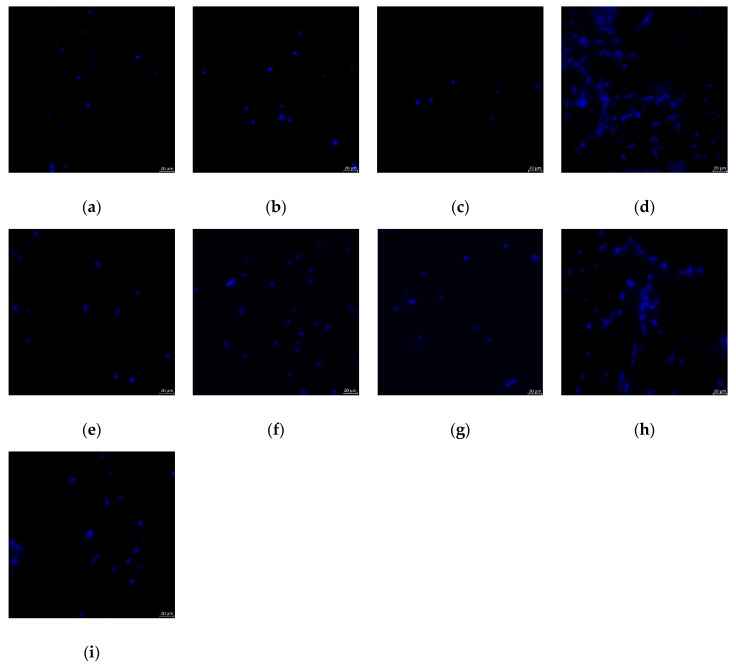
Exemplary epifluorescence microscopic images of *C. albicans* cells on specimens in each group: (**a**) PMMA + CS-G 0.1%, (**b**) PMMA + CS-G 0.3%, (**c**) PMMA + CS-G 1%, (**d**) PMMA + CS-G 3%, (**e**) PMMA + CS-HCl 0.1%, (**f**) PMMA + CS-HCl 0.3%, (**g**) PMMA + CS-HCl 1%, (**h**) PMMA + CS-HCl 3%, (**i**) PMMA (control); polymethylmethacrylate (PMMA), chitosan glutamate (CS-G), chitosan-hydrochloride (CS-HCl).

Figure 3 shows exemplary epifluorescence images of the evaluated surface areas. A larger number of fungal cells could be identified on specimens modified with 3% of CS-HCl and CS-G.

## 3. Materials and Methods 

### 3.1. Chitosan(CS)-Salts 

CS-salts (Chitoceutisals) were purchased from Heppe Medical Chitosan GmbH (Halle, Germany). The chitosan-hydrochloride (CS-HCl) had the following specifications: deacetylation degree (DDA): 85.7%, chlorides: 16%, molecular weight (MW): 30–400 kDa. Chitosan-glutamate (CS-G) with DDA 91.4% and MW 30–600 kDa were used.

### 3.2. Preparation of CS-Salt Modified PMMA Specimens

Cylindrical polymethyl methacrylate (PMMA) specimens (*n* = 72, Ø12.75 mm, height 6 mm) were manufactured (Palapress pink, Hereus, Hanau, Germany). CS-HCl (*n* = 32) and CS-G (*n* = 32) in concentrations of 0.1%, 0.3%, 1% and 3% (*n* = 8 each salt and concentration) were added to PMMA resin. The PMMA resin was prepared according to the manufacturer’s instructions. For the control group (*n* = 8), the PMMA resin mixture was prepared by mixing 7 mL of a monomer liquid with 10 g of PMMA powder for 15 to 30 s at room temperature (23 °C), and polymerizing for 20 min at 55 °C under 2.3 bar pressure (rm-Dental Acryclave, rm-Dental, Memmelsdorf, Germany). For the CS-salt-modified specimens 0.017 g, 0.05 g, 0.17 g, and 0.5 g of each CS-salt were added to 10 g PMMA powder and mixed for 5 min. The PMMA/CS-salt mixture was added to 7 mL of monomer liquid and mixed for 15 to 30 s at room temperature (23 °C), then polymerized as described for the control group. To produce cylindrical specimens with a standardized size, a metal casting mold was used. To standardize and simulate denture roughness at the palatal site, all specimens were prepared first with 800-grid, followed by 1200-grid, sandpaper (Struers SiC Foil #800 und #1200, Struers RotoPol-22, Struers GmbH, Copenhagen, Denmark).

### 3.3. Roughness Measurement

The specimen roughness was measured by contact profilometry (Hommel Etamic W 20, waveline 20 measuring station, Jenoptik, Schwenningen, Germany) before the antifungal tests. The linear traverse unit was positioned parallel to the specimen surface, aligned, and adjusted. The roughness values (Ra) were measured at 3 sites per specimen and the arithmetical average values per specimen were calculated (Excel 2016, Microsoft Office, Microsoft Corporation, Redmond, WA, USA). 

### 3.4. Antifungal Test

#### 3.4.1. Candida Cell Suspension

A loopful of *C. albicans* cultures (ATCC 2091, Manassas, VA, USA) was cultivated on a sabouraud agar plate (glucose 20 g/L, agar-agar 12 g/L, peptone from casein 5 g/L, peptone from meat 5 g/L, pH 5.7 ± 0.2). One colony was transferred from the agar into dextrose broth (containing penicillin 100 µg/mL and streptomycin 100 µg/mL) and incubated. The incubated cells were centrifuged (500× *g* for 4 min), suspended, diluted, and standardized with a cell density of 8 × 10^5^ cells/mL for optical density measurement and 2–3 × 10^7^ cells/mL for the biofilm assay, controlled by a spectrophotometer (VWR UV-1600PC, VWR International, Leuven, Belgium) at an absorbance of 1.1 at 600 nm.

#### 3.4.2. Chitosan-Salt Solution

CS-salts were dissolved in 5 mL of sterile aqua dest. in concentrations 6% (60mg/mL), 2% (20mg/mL), 0.6% (6 mg/mL) and 0.2% (2mg/mL) for 24 h at 37 °C in a shaker (100 rpm) after that 5 mL of *C. albicans* suspension were added and incubated at 37 °C in a shaker (100 rpm) for over 10 h. Following this, the final concentrations of CS-salts in the tested groups (3%, 1%, 0.3% and 0.1%) were evaluated.

#### 3.4.3. Optical Density Measurement

Optical density was measured in a spectrophotometer (VWR UV-1600PC, VWR International, Leuven, Belgium) at an absorbance of 1.1 at 600 nm. For each CS-salt group, the test was repeated (*n* = 4) with constant optical density measurements. 

#### 3.4.4. Biofilm Assay and Fungal Cell Count

The PMMA specimens were cleaned in aqua dest. for 10 min using an ultrasonic bath (Sonorex Digital 10 P, Brandelin electronic, Berlin, Germany), placed in a 24-well plates (Corning 24 Well Cell Culture Cluster, Corning Incorporated, Corning, NY, USA) and stored for 7 days in aqua dest. at 37 °C for residual monomer release [29]. After 7 days of storage in aqua dest., the specimens were disinfected with 70% ethanol for 60 s and stored 90 min in sterile aqua dest. Primarily, an experimental pellicle was formed. Therefore, the specimens were incubated for 2 h at 37 °C with centrifugated (4000× *g* for 10 min) and microfiltered (0.2 µm filter) human unstimulated saliva. After pellicle formation, the specimens were placed in new 24-well plates and the candida cell suspension was added. The specimens were incubated in a shaker for 2 h at 37 °C, then the candida suspension was washed out twice with phosphate-buffered saline (PBS) and dextrose broth was added again to each well. For biofilm formation, the specimens were incubated in a shaker (100 rpm) for 24 h at 37 °C. Single-species *C. albicans* biofilm developed on the specimens. After biofilm formation, the specimens were washed with PBS, fixed with methanol for 10 min, and stored in 4% formaldehyde at 4 °C until staining with Calcofluor and microscopic evaluation. The candida cells adhering to the specimens were stained with Calcofluor (Fluka Calcofluor White Stain, Fluka Analytical, München, Germany). For analyzing the number of fungal cells adhering to the specimen surface, the epifluorescence microscopy method was used [39,40]. Light filters (BP 381-399, FT 416, LP 430-490, Carl Zeiss, Jena, Germany) were used, and the counts were made at a magnification of 20× (Zeiss Axioplan, 451889, Carl Zeiss, Jena, Germany). In 10 randomized fields of 0.0576 mm^2^ per sample, the number of fungal cells was counted (Zeiss AxioVision 4.8.2, Carl Zeiss, Jena, Germany). For statistical analysis, the values were converted in the number of fungal cells per cm^2^. 

### 3.5. Statistical Analysis

For statistical analysis, the number of fungal cells per cm^2^, and the analysis of relative growth (calculated as described by Seyfahrt et al. 2008) were used [37]. A Shapiro–Wilk test was used to prove the normal distribution of the data. For the analysis of cell counts and roughness, Kruskal–Wallis and U-tests were used. For the analysis of relative growth, t-tests were conducted by the use of SPSS software (IBM SPSSStatistics, V 25, IBM, Armonk, NY, USA) for Windows. The *p* values were adjusted using Bonferroni–Holm correction [41]. The level of significance was set at α = 0.05.

## 4. Discussion

Fungal growth was reduced by chitosan (CS)-salts. Nevertheless, except for one group, no significant decrease in fungal cell counts were found between the denture base material modified with CS-salt powders and the unmodified standard material (control). The CS-salts showed good antifungal activity, but adding them to denture base material makes them ineffective. The increased roughness in almost all tested groups, caused by the addition of CS-salts, could promote fungal attachment and biofilm formation.

Previous studies showed promising results regarding the antimicrobial and antibiofilm activity of CS [18,22,31,32,37,42,43]. These results could be confirmed for CS-salts, but not for polymethyl methacrylate (PMMA) modified by them. 

With similar approaches to mixing CS with dental materials as tissue conditioners or light-cured resins, Saeed at al., Sadeghi Ardestani et al. and Lee et al. showed good antifungal or antibiofilm effects of CS against *C. albicans* [31,32,42]. Saeed at al. tested CS and CS-oligosaccharides incorporated into tissue conditioner [32], while Lee at al. tested CS and quaternized CS [42]. Sadeghi Ardestani et al. used CS-nanoparticles mixed with light-cured resin [31]. These results could not be confirmed in the present study. These differences could be explained by the use of diverse CS formulations and their particle size or by different evaluation methods. [40,44].

In previous studies, CS-G or CS-HCl showed antibacterial [38] and CS-HCl antifungal activity against *C. albicans* [37]. This antifungal activity could be confirmed in the present study.

CS was also used in other approaches to prove its antifungal and antibiofilm activity, particularly as CS solution, CS mouthwash, or CS coatings for use in the treatment of denture stomatitis (DS). The described approaches showed promising results and confirmed the antifungal and antibiofilm activity of CS [45,46,47].

Contrary to the present study, the biofilm formation of *Candida* spp. was decreased by about 70% in adhesion and 80% in the mature phase by CS solution [48]. The authors described CS as a promising anti-candidiasis agent [45].

Furthermore, CS caused a delay in *C. albicans* biofilm formation and defects in biofilm morphology, due to the inhibition of cell growth [49].

Contrary to the present study, Ramana et al. showed that CS/hyaluronic acid (HA) coatings exhibit inherent antifungal activity against planktonic *C. albicans* after 6 h and reduce biofilm formation on coated surfaces [50]. The biofilm observed was less dense and robust than that observed in groups without CS/HA coating. Moreover, surfaces coated with CS showed good antibiofilm properties. CS coatings interrupt bacterial and fungal (*C. albicans*) biofilm formation, probably by the permeabilization of microbial cells in contact with the coated surface. One possible method of action of CS against fungi is the interaction between cationic-loaded CS molecules and negatively charged macromolecule residues on the fungal cell membrane, leading to leakage in the membrane and apoptosis of the cell [42]. Nevertheless, the dead cells remain on the CS-coated surface [51].

A similar process could occur in the present study, as the remained dead cells cover the CS-salts particles embedded in the material and so decreased their antifungal action. Moreover, the particles were not dissolved and could have not enough polycationic groups. In the present study, Calcofluor staining was chosen to quantify the complete amount of adhered and biofilm-forming cells. Calcofluor stains the cell walls of yeast, independent of the metabolic state of the cell [52]. This could explain the higher amount of candida cells on specimens in the present study, as both dead and live cells were counted. The adhesion of new yeast cells from the medium on a layer of dead cells cannot be excluded. This is one of the limitations of this study and further studies with other staining methods are needed.

Surface roughness has a direct influence on the initial adherence of micro-organisms, on the biofilm formation, and the colonization with *Candida* spp. [1] Rough surface increases yeast counts because of the higher possibility of microorganism adhesion, attachment, and retention in the surface irregularities, as well in protection against shear forces [1]. In the literature, a roughness threshold (Ra) of 0.2 µm is described; values below influence the microbial adhesion negligibly [1,53]. In this study, a worst-case baseline roughness above the threshold of 0.2 µm was chosen. The surface roughness increased by adding CS-salts to the denture base material in this study. Waltimo et al. showed that the interface between denture base material and additives such as glass fibers increases the adherence of fungal cells to the material surface [54]. This interface could also be the weak point of the specimens tested in this study. The approach of the development of antifungal surfaces by addition of CS-salt powder resulted in increased roughness, a crucial factor in microorganism attachment, as the surface roughness affects the fungal cell attachment and biofilm formation.

A further, limitation of this study is the use of monospecies biofilm as a very simple model far from the conditions in the oral cavity [55]. Nevertheless, *C. albicans* is the most prevalent and predominant *Candida* spp. associated with DS [6]. The specimen design and experiment parameters could also influence the results [56,57].

The authors concluded that the approach of mixing CS-salt powders to denture base material is inferior for treatment or prevention of DS in comparison with other approaches such as CS gels, CS mouthwash, or CS denture base coatings.

In our study, good antifungal activity was shown for CS-salt solutions. On the other hand, no inhibition of adherence or decreased biofilm formation of *C. albicans* to CS-salt-modified PMMA surface could be shown. Furthermore, the aesthetic properties of the material change unfavorably because the superficial whitish particles of the chitosan salts, which are scattered throughout the bulk of the resin material, are visible on the resin surface due to the addition of CS-G and CS-HCl. Taking the above into consideration, the studied concept of modifying denture base materials with CS requires improvement and further investigation.

## 5. Conclusions

Within the limitations of this in vitro study, CS-salts have antifungal activity, but the modification of denture base materials by adding CS-salt powders to PMMA increased the roughness significantly and did not result in antifungal, antibiofilm, or anti-adherent effects. Modification of denture base material through mixing with CS-salt powders does not appear to be a first-choice option to develop materials with antifungal, antibiofilm, anti-adherent properties for use in the prevention or treatment of denture stomatitis.

## Figures and Tables

**Table 1:** Descriptive data for *C. albicans* cell counts per 1 cm^2^.

Group	Mean	SD	Median	Min	Max
PMMA + CS-G 0.1%	1.79 × 10^4^	1.03 × 10^4^	1.90 × 10^4^	1.00 × 10^3^	3.50 × 10^4^
PMMA + CS-G 0.3%	4.48 × 10^4^	1.00 × 10^5^	1.05 × 10^4^	1.00 × 10^3^	2.93 × 10^5^
PMMA + CS-G 1%	1.26 × 10^4^	1.89 × 10^4^	3.50 × 10^3^ *^A^	2.00 × 10^3^	5.70 × 10^4^
PMMA + CS-G 3%	3.76 × 10^5^	1.85 × 10^5^	3.41 × 10^5^	1.52 × 10^5^	7.20 × 10^5^
PMMA + CS-HCl 0.1%	2.83 × 10^4^	3.34 × 10^4^	2.00 × 10^4^	8.00 × 10^3^	1.10 × 10^5^
PMMA + CS-HCl 0.3%	2.88 × 10^4^	1.28 × 10^4^	2.90 × 10^4^	8.00 × 10^3^	4.60 × 10^4^
PMMA + CS-HCl 1%	1.30 × 10^4^	1.04 × 10^4^	1.25 × 10^4^ *	2.00 × 10^3^	3.00 × 10^4^
PMMA + CS-HCl 3%	7.43 × 10^4^	4.39 × 10^4^	6.85 × 10^4^	2.90 × 10^4^	1.69 × 10^5^
PMMA (control)	5.08 × 10^4^	4.48 × 10^4^	3.85 × 10^4^ *^A^	1.00 × 10^4^	1.31 × 10^5^

Polymethylmethacrylate (PMMA), chitosan glutamate (CS-G), chitosan-hydrochloride (CS-HCl), standard deviation (SD), minimum (min), maximum (max), * indicate significant difference to control (*p* -values row ≤ 0.05), ^A^ superscript letter indicates significant difference to control (*p*-values adjusted ≤ 0.05). Group PMMA+CS-G 3% was not considered in the statistical analysis.
